# Upregulated Vanins and their potential contribution to periodontitis

**DOI:** 10.1186/s12903-022-02583-7

**Published:** 2022-12-17

**Authors:** Weijun Yu, Shucheng Hu, Ruhan Yang, Lu Lin, Chuanyuan Mao, Min Jin, Yuting Gu, Guanglong Li, Bin Jiang, Yuhua Gong, Eryi Lu

**Affiliations:** Department of Stomatology, Renji Hospital, Shanghai Jiao Tong University School of Medicine, Shanghai, 200127 China

**Keywords:** Vanin, Periodontitis, Gingival tissue, Neutrophil

## Abstract

**Background:**

Although Vanins are closely related to neutrophil regulation and response to oxidative stress, and play essential roles in inflammatory diseases with clinical significance, their contribution to periodontitis remains to be determined. This research was designed to assess the expression of Vanins in human gingiva, and to define the relationship between Vanins and periodontitis.

**Methods:**

Forty-eight patients with periodontitis and forty-two periodontal healthy individuals were enrolled for gingival tissue sample collection. Expression levels of *VNN1*, *VNN2* and *VNN3* were evaluated by RT-qPCR and validated in datasets GSE10334 and GSE16134. Western blot and immunohistochemistry identified specific proteins within gingiva. The histopathological changes in gingival sections were investigated using HE staining. Correlations between Vanins and clinical parameters, PD and CAL; between Vanins and inflammation, *IL1B*; and between Vanins and MPO in periodontitis were investigated by Spearman's correlation analysis respectively. Associations between *VNN2* and indicators of neutrophil adherence and migration were further validated in two datasets.

**Results:**

Vanins were at higher concentrations in diseased gingival tissues in both RT-qPCR and dataset analysis (*p* < 0.01). Assessment using western blot and immunohistochemistry presented significant upregulations of VNN1 and VNN2 in periodontitis (*p* < 0.05). The higher expression levels of Vanins, the larger the observed periodontal parameters PD and CAL (*p* < 0.05), and *IL1B* (*p* < 0.001). Moreover, positive correlations existed between VNN2 and MPO, and between *VNN2* and neutrophil-related indicators.

**Conclusion:**

Our study demonstrated upregulation of Vanins in periodontitis and the potential contribution of VNN2 to periodontitis through neutrophils-related pathological processes.

**Supplementary Information:**

The online version contains supplementary material available at 10.1186/s12903-022-02583-7.

## Background

Periodontitis is a widely prevalent infectious inflammatory disease of the periodontal tissues in response to the invasion of bacterial pathogens [1, 2]. Severe periodontitis affects 10–15% of population, while 40–60% of adults suffer from moderate periodontitis [3]. The imbalance between proinflammatory and anti-inflammatory cytokines and deleterious effects during the host’s response against bacterial infection could lead to irreversible periodontium destruction, including periodontal pockets formation and alveolar bone resorption [4–7].

During the pathological process of periodontitis, neutrophils not only play protective roles, such as helping to maintain periodontal tissue homeostasis, but may also lead to destructive effects, such as affecting inflammatory bone loss [8]. Neutrophils can be recruited quickly to the attacked site in acute inflammation, whereas they are also involved in chronic inflammatory diseases [9]. In chronic periodontitis, a larger number of longer-lived hyperactivated neutrophils promote the lymphocyte-rich periodontium lesion, resulting in aggravating periodontal tissue destruction [9]. In addition, hyperactive neutrophils promote reactive oxygen species production, leading to dysregulated metabolites of lipid peroxidation, damage of DNA and proteins during pathological process of periodontal lesions [10]. Factors regulating neutrophil functions were therefore hypothesized to affect periodontitis pathological process [9, 11].

Vanins (VNNs) are pantetheinase enzymes, including three members VNN1, VNN2, and VNN3, which mediate hydrolyzation of pantetheine in Coenzyme A circulation [12]. Numerous studies have revealed pivotal roles of Vanins in fatty acid metabolism, oxidative stress, cell migration and inflammatory response [13–15]. VNN1 was first identified as a regulator of adhesion of thymocytes and thymus homing, and reported to affect oxidative stress [16]. VNN2 is mainly distributed on human neutrophils and monocytes, with secretory vesicles as the reservoir of intracellular VNN2 [17, 18]. Working as a modulator of Mac-1 (CD11b/CD18), VNN2 was reported to regulate β2 integrin-mediated cell adhesion, cell migration, and motility of neutrophils [16]. Recent studies indicated that expression levels of Vanins were abnormal in diverse diseases, especially inflammatory diseases, including diabetes, sepsis, rheumatoid arthritis and inflammatory bowel disease [19–23]. The dysregulated Vanins played significant roles of biomarkers in disease diagnosis and prognosis [24]. In view of these findings, we would consider the potential role of Vanins in periodontitis.

A recent transcriptomic analysis of circulating lymphocytes and monocytes indicated that the VNN1 gene was upregulated in patients suffering from type II diabetes mellitus, periodontitis, and dyslipidemia simultaneously in contrast with normal individuals [25]. However, the expression profile of Vanins in periodontitis on both the genetic and protein levels has not been reported, and it was unclear how Vanins contribute to periodontitis. Therefore, the study was mainly aimed to analyze the relationship between Vanins and periodontitis. Comparison of the expression of Vanins in human gingival tissues was conducted between patients with periodontitis and periodontal healthy individuals. In addition, the Vanins expression levels were hypothesized to associate with periodontitis on the aspect of clinical parameters, the severity of inflammation and neutrophil response.

## Methods

### Study design and participants

The study was performed between February 2022 to October 2022 at the Department of Stomatology, Renji Hospital affiliated to Shanghai Jiao Tong University School of Medicine, following the STROBE guidelines (Supplementary File 1). Approval from the Institutional Review Board was obtained before subject enrollment. The study design was developed according to Helsinki Declaration (KY2021-196-B). The research group obtained the written informed consent from all participants enrolled.

Patients diagnosed with periodontitis or impacted mandibular third molars were considered to be potentially eligible. The screening was conducted to confirm eligibility after clinical examination and interviews of demographic information. After the screening study, the subjects were enrolled into the study with informed consent. According to the classification of periodontal conditions updated in 2017, inclusion criteria for the periodontal healthy groups were as follows: (1) probing depth (PD) ≤ 3 mm, bleeding on probing (BOP) < 10% and no clinical attachment loss (CAL), no history of periodontitis and no history of pericoronitis; (2) aged between 18 and 65 [26]. Inclusion criteria for the periodontitis group were as follows: (1) diagnosed with stage II-IV periodontitis, whose maximum PD ≥ 5 mm, CAL ≥ 3 mm at the most severe site, bone loss ≥ 15% in radiographic examinations; (2) needed periodontal flap surgery in the mandibular posterior region; and (3) aged between 18 and 65 [27]. Subjects were excluded with (1) smoking history, (2) pregnant, lactating, or menopausal status, (3) taking any medication which could affect periodontal healthy conditions over the past quarter, or (4) accompanied with systemic diseases relating to periodontal lesions, including but not limited to diabetes, cardiovascular diseases, systemic lupus erythematosus and chronic renal diseases [6]. In total, this study enrolled 48 periodontitis patients and 42 periodontal healthy individuals as the flow diagram presented (Supplementary Fig. 1).

### Sample collection

The demographic information was obtained by interviewers, and the clinical characteristics were collected by a specialist of periodontology when screening the eligibility (Table 1). The clinical parameters, including PD and CAL, were performed at six sites (mesial-buccal, mid-buccal, distal-buccal, mesial-lingual, mid-lingual, and distal-lingual) and the deepest site was chosen for clinical parameters collection. Gingival tissue samples were collected from 48 periodontitis patients during periodontal flap surgery, in which the inner margin of the flap was trimmed. For periodontal healthy group, tissue samples were obtained from 42 donors who had their impacted teeth extracted. All attached gingival tissue samples were from mandibular posterior regions, comprising the epithelial and connective tissue. The collected samples were rinsed with 0.9% normal saline and kept at − 80 °C for further experiments, while others were fixed with 4% Paraformaldehyde for further Hematoxylin–eosin (HE) staining and IHC. In total, 48 periodontitis and 42 healthy samples were collected, of which 30 pairs were used for RT-qPCR, six pairs were used for western blot, and the other 12 periodontitis and six healthy samples were used for histological analysis.

**Table 1:** Demographic information and clinical characteristics of periodontal healthy individuals and patients with periodontitis enrolled in this study

Characteristics	Periodontal healthy (*n* = 42)	Periodontitis (*n* = 48)
Age (years)	30.81 ± 4.70	33.25 ± 5.22
Gender (Male/Female)	21/21	24/24
Number of Tooth loss	0.00 ± 0.00	0.40 ± 0.82***
PD (mm)	2.26 ± 0.54	6.75 ± 1.41****
CAL (mm)	0.00 ± 0.00	6.06 ± 2.04****
BOP (%)	7.21 ± 1.76	72.29 ± 14.37****

Data are expressed as mean ± SD. Statistical significance is indicated as *** *p* < 0.001, and **** *p* < 0.0001

*PD* Probing depth, *CAL* Clinical attachment loss, *BOP* Bleeding on probing

### RT-qPCR

Gingival tissue samples of periodontitis (*n* = 30) and periodontal healthy (*n* = 30) donors were utilized for RT-qPCR as previously reported [6]. Total RNA was isolated with TRIzol Reagent (Invitrogen, Carlsbad, CA, USA), and was later qualified and quantified. Using PrimeScript RT Master Mix (Takara Bio, Otsu, Shiga, Japan), RNA was reverse-transcribed into cDNA according to manufacturer's protocol. RT-qPCR was performed in usage of FastStart Universal SYBR Green Master Mix (Roche, Nutley, NJ, USA) on the available QuantStudio7 Flex Real-Time PCR System (Applied Biosystems, Foster City, CA, USA). The relative mRNA expression levels of detected *VNN1, VNN2*, *VNN3*, and *IL1B* were normalized to the housekeeping gene *GAPDH* in the 2^-ΔCT method. Experiments designed were all repeated at least in triplicate. The primers from Sangon Biotech (Shanghai, China) were available in Table 2 with details of primer design.

**Table 2:** Primer sequences for RT-qPCR validation

Gene	Forward primer (5’ → 3’)	Reverse primer (5’ → 3’)
*VNN1*	TCTGCAGTGGTGAACTGGAC	GTCAAATGCCCCTAGAGCGT
*VNN2*	GTGCTACTTACCGAAATTCATC	TTTACCAAACGCCCATCTT
VNN3*	GATCATTCTAAGTGGGAGTCA	CGTCCATCTCTTGAAATCTCA
*IL1B*	AGCTACGAATCTCCGACCAC	CGTTATCCCATGTGTCGAAGAA
*GAPDH*	GGAAGATGGTGATGGGATT	GGATTTGGTCGTATTGGG

^*^VNN3, VNN3P Vanin 3, pseudogene (Homo sapiens)

### Validation on GEO datasets

The periodontitis datasets were searched within the datasets registered in the GEO of the accessible National Center for Biotechnology Information (NCBI) (www.ncbi.nlm.nih.gov/geo/). Two datasets, GSE10334 and GSE16134, which examined gene expression profiles in the diseased and healthy human gingival sites, were obtained. For the GSE10334 dataset, the transcriptomes of 183 diseased and 64 periodontal healthy gingival sites were reported. For the GSE16134 dataset, the gene expression profiling of human periodontitis was presented with a total number of 310 gingival sites (241 diseased and 69 healthy sites). Both GSE10334 and GSE16134 were from the platform GPL570. To validate Vanin expression levels and their correlation with inflammation, relative expression levels of *VNN1, VNN2, VNN3* and *IL1B* were selected for further analysis. The correlation between Vanins and neutrophils, especially in regards to the previously reported β2 integrin-dependent neutrophil adherence and migration, was conducted using relative expression levels of subunits of α/β integrins and ligands of β2 integrins on these two datasets [28, 29]. The average value was calculated if there were multiple probes for the same gene.

### Western blot

Six pairs of gingival tissue samples were used in western blot. As reported in the previous article of research group, the total protein was extracted by RIPA Lysis Buffer (Beyotime, Shanghai, China) with 1% PMSF (Beyotime, Shanghai, China) on ice from gingival samples of six periodontitis donors and six normal controls [6]. The Thermo Scientific Pierce BCA Protein Assay Kit was used to measure protein concentrations. Based on the protein concentration, protein solutions were diluted, denatured, and loaded onto sodium dodecyl sulfate–polyacrylamide gels. The polyvinylidene fluoride membrane loaded proteins from gels and were further blocked with 5% bovine serum albumin (BSA). Primary antibodies against VNN1 (ab205912, Abcam), VNN2 (#39964, Signalway Antibody) and GAPDH (5174 s, Cell Signaling Technology) were used for overnight membrane treatment at 4 °C. Next, the 1 × phosphate buffered saline (PBS) with Tween detergent (PBST) was used to wash the membranes, which were later treated with Anti-rabbit IgG, HRP-linked Antibody (Cell Signaling Technology, Danvers, MA, USA). PBST washed the membranes again. The Chemiluminescence Reagents (Millipore, Billerica, MA, USA) helped visualize immunoreactive bands. Quantitative analysis of blots was conducted on ImageJ software.

### Sections preparation

The gingival tissues of six periodontal healthy individuals and 12 periodontitis patients were fixed with 4% Paraformaldehyde for more than 24 h at 4 °C. Specimens were embedded in paraffin and cut into 4-μm sections. The section was later dewaxed with routine xylene, followed by dehydration. The sections were prepared for HE staining and IHC assay.

### HE staining

Sections of gingival samples were treated with Hematoxylin solution, followed by Hematoxylin differentiation solution treatment as well as Hematoxylin Scott Tap Bluing step. Next, the sections were dehydrated and stained with Eosin dye and sealed with neutral gum. The histopathological changes of human gingival tissues were observed by researchers under an optical microscope to define the periodontal status. The results of HE staining were evaluated and recognized by oral pathologists as well.

### Immunohistochemistry assay

To specifically detect target antigens, sections were blocked with 3% BSA after antigen retrieval. After removal of excess liquid, gingival sections were incubated with a rabbit antibody against VNN1 (1:500, ab205912; Abcam), a rabbit antibody against VNN2 (1:500, #39964; Signalway Antibody) and a rabbit antibody against MPO (1:1000, GB11224; Servicebio) overnight according to protocols. Sections, which were washed in PBS for three times, were incubated with biotinylated goat anti-rabbit IgG secondary antibody for 50 min at room temperature. The positive is brownish yellow after DAB chromogenic reaction. After nucleus counterstaining, dehydration and mounting, the sections were scanned under a microscope. The positive area percentage was analyzed as a positive rate in quantitative analysis. Analysis of high-power fields was conducted after random selection to determine percentages of positive areas and the average optical density (AOD).

### Statistical analysis

In this study, all data were statistically analyzed with GraphPad Prism 8.0 and reported mean ± standard deviation (SD). Mann–Whitney test was used to compare two groups of independent samples. Correlations between Vanins expression levels and the periodontal clinical parameters, including PD and CAL, were analyzed according to Spearman's correlation analysis. Correlation analysis was further conducted between Vanins and *IL1B* expression based on RT-qPCR and datasets, between Vanins and MPO based on IHC, and between *VNN2* and neutrophil-related indicators based on datasets. * *p* < 0.05 was considered to be statistically significant. Other forms were expressed as ** *p* < 0.01, *** *p* < 0.001 and **** *p* < 0.0001.

## Results

### Upregulated expression levels of Vanins in periodontitis gingival tissue samples

**Fig. 1:**
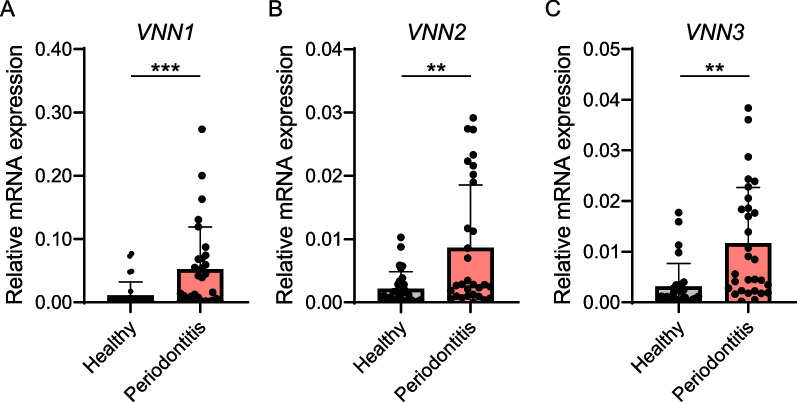
Upregulation of VNNs in periodontitis in RT-qPCR. Genetic expression levels of *VNN1* (**A**), *VNN2* (**B**), and *VNN3* (**C**), in gingival tissues from periodontal healthy (*n* = 30) and periodontitis (*n* = 30) subjects were evaluated. The data were presented in the form of mean ± SD. Significance was defined in the figure as ** *p* < 0.01 and *** *p* < 0.001

RT-qPCR was used to determine the relative expression levels of *VNN1, VNN2* and *VNN3*. All three were significantly upregulated in diseased gingival tissues from periodontitis (*n* = 30) in comparison with the periodontal healthy samples (*n* = 30) (Fig. 1).

**Fig. 2:**
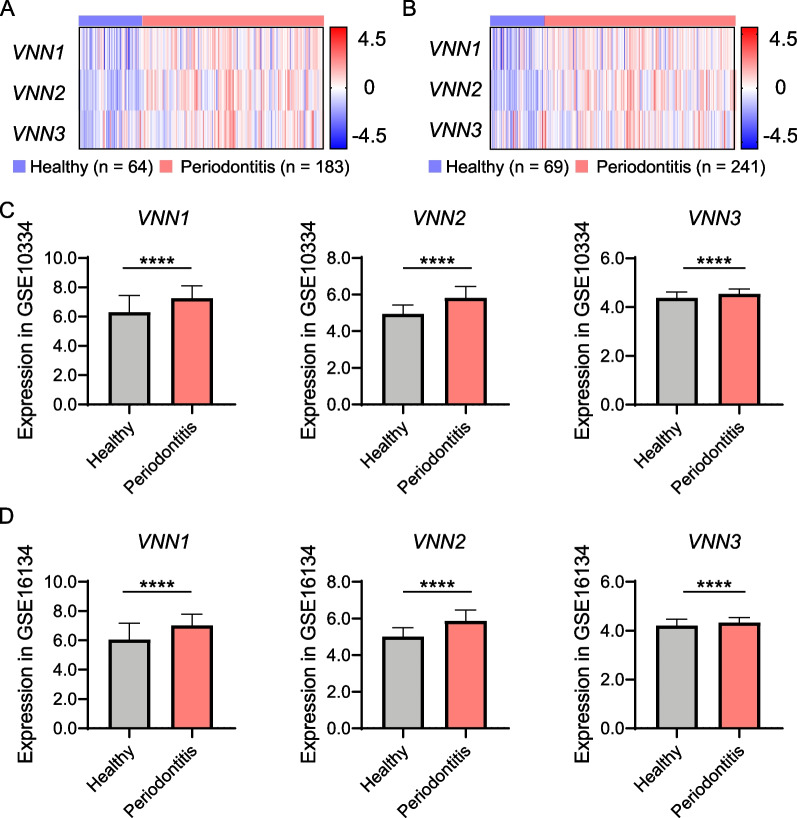
Validation of gene expression levels of VNNs in datasets. **A**, **C** VNNs in healthy (*n* = 64) and periodontitis (*n* = 183) sites in dataset GSE10334 were presented. **B**, **D** VNNs in healthy (*n* = 69) and periodontitis (*n* = 241) sites in dataset GSE16134 were presented. Z-score was analyzed and enrolled in the establishment of heatmaps. Significance was defined in the figure as **** *p* < 0.0001

Two datasets, GSE10334 and GSE16134, were studied to further validate Vanins upregulation in human periodontitis. Consistent with the RT -qPCR result from our samples, the expression levels of *VNN1, VNN2,* and *VNN3* in the datasets were significantly higher in diseased gingival tissues (*p* < 0.0001) (Fig. 2). Generally speaking, expression levels of *VNN1, VNN2* and *VNN3* were higher in gingival tissues from periodontitis patients at the genetic level in both RT-qPCR and GEO dataset analysis. For *VNN3* was previously reported to be a pseudogene for the frame shift owing to the absence of one nucleotide, we decided to only study VNN1 and VNN2 in the proceeding experiments.

**Fig. 3:**
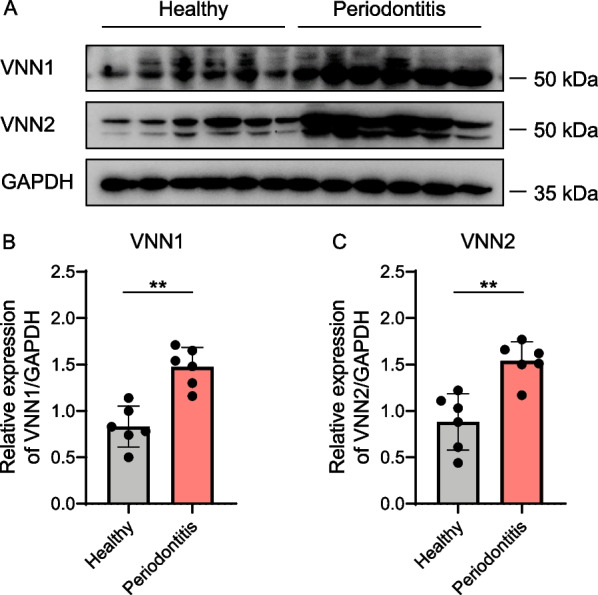
Upregulation of VNN1 and VNN2 in periodontitis in western blot. **A** Protein expression levels of VNN1 and VNN2 in gingival tissue samples from periodontal healthy (*n* = 6) and periodontitis (*n* = 6) subjects were evaluated. **B** Relative expression level of VNN1 protein when normalized to GAPDH. **C** Relative expression level of VNN2 protein when normalized to GAPDH. Significance was defined in the figure as ** *p* < 0.01

As immunoreactive bands and the matched quantitative assessment shown in Fig. 3, VNN1 and VNN2 presented significantly higher concentrations in diseased gingival tissues (*n* = 6) on the protein level in comparison with periodontal healthy ones (*n* = 6) (*p* < 0.01). The upregulation of VNN1 and VNN2 protein levels in western blot was consistent with the results of genetic expression.

### Expression and involvement of VNN1 and VNN2 in the histopathological changes of gingival tissues with periodontitis

**Fig. 4:**
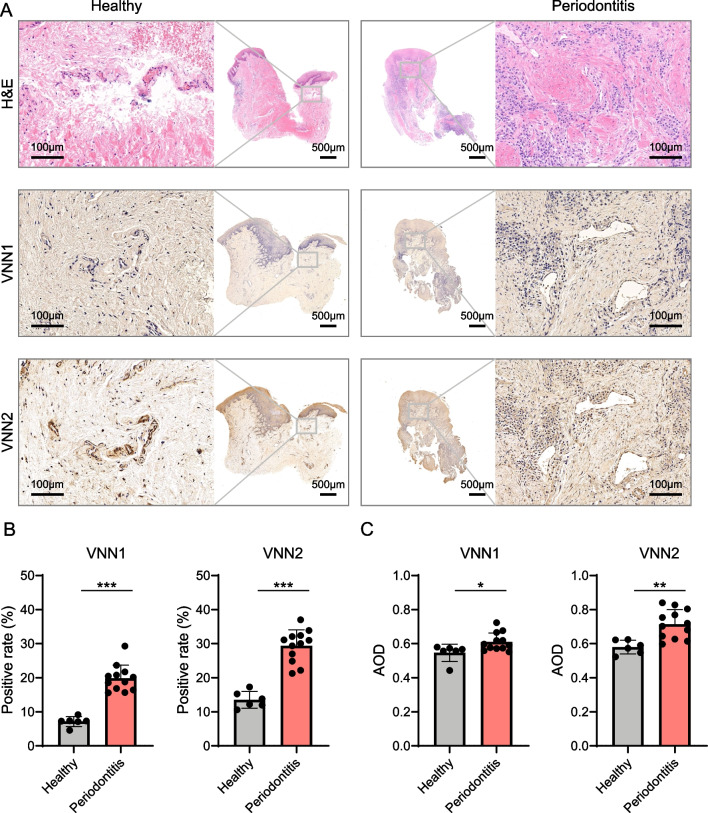
VNN1 and VNN2 were at higher concentrations and got involved in the histopathological changes of gingival tissues with periodontitis. **A** HE staining and immunohistochemical staining of VNN1 and VNN2 in gingival tissues of periodontal healthy individuals and patients with periodontitis. Positive expression areas of VNN1 and VNN2 were brownish yellow in the connective tissue. **B** The positive area percentage of VNN1 and VNN2 in the gingiva from periodontal healthy individuals (*n* = 6) and patients with periodontitis (*n* = 12). **C** The quantitative analysis of AOD of VNN1 and VNN2 in the gingiva from healthy (*n* = 6) and periodontitis (*n* = 12) subjects. Significance was defined in the figure as * *p* < 0.05, ** *p* < 0.01, and *** *p* < 0.001. AOD, average optical density

HE staining demonstrated histological characteristics in diseased and periodontal healthy gingival tissues (Fig. 4A). The structure of healthy gingival tissues was normal with clear epithelial and connective tissue layers, and free of inflammation. Whereas in the diseased gingival tissues, a large number of immune cells infiltrated the connective tissues after HE staining. Positive expressions of VNN1 and VNN2 were presented in the connective tissue regions, as indicated by IHC (Fig. 4A). The positive rates of VNN1 and VNN2 were significantly higher in inflamed gingival tissues than in healthy samples (*p* < 0.001), as shown in Fig. 4B. The average optical density (AOD) of VNN1 and VNN2 was significantly higher in periodontitis gingival tissues than periodontal healthy samples as well (*p* < 0.05) (Fig. 4C). The results supported that VNN1 and VNN2 were enriched in gingival tissues affected by periodontitis, especially in the part of connective tissues.

### Clinical significance of Vanins in human periodontitis

The association between Vanin family members, *VNN1* and *VNN2*, and periodontal parameters, PD and CAL, were investigated according to Spearman’s correlation analysis (Table 3). The expression level of *VNN1* was significantly positively correlated to PD (R = 0.4498, *p* = 0.0126, moderate) and CAL (R = 0.5867, *p* = 0.0007, moderate). In addition, the relative expression level of *VNN2* was significantly positively related to PD (R = 0.6218, *p* = 0.0002, moderate) and CAL (R = 0.5912, *p* = 0.0006, moderate). The positive correlations indicate potential roles Vanins play in periodontitis, and that *VNN2* displays a stronger correlation with clinical parameters than *VNN1*.

**Table 3:** Correlation analysis of overexpressed VNNs and periodontal parameters

	PD (mm)		CAL (mm)	
	**R**	***p***	**R**	***p***
***VNN1***	0.4498*	0.0126	0.5867***	0.0007
***VNN2***	0.6218***	0.0002	0.5912***	0.0006

Statistical significance was indicated as * *p* < 0.05, and *** *p* < 0.001

*PD* Probing depth, *CAL* Clinical attachment loss

### Correlation between upregulated Vanins and inflammation in periodontitis

**Fig. 5:**
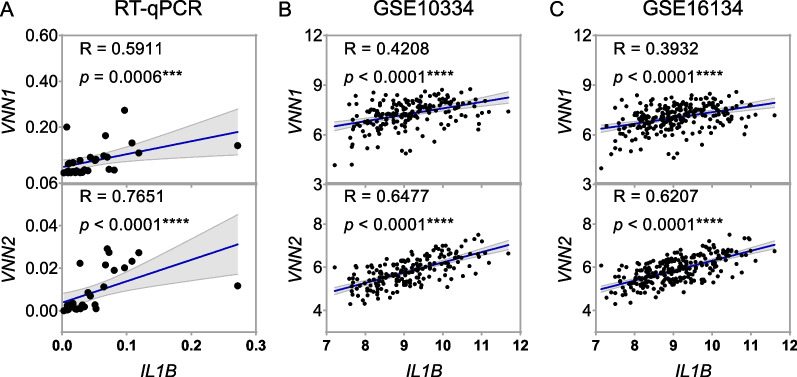
Upregulated VNNs were positively correlated with inflammation. **A** Correlation between expression levels of *VNN1* and *IL1B*, and between expression levels of *VNN2* and *IL1B*, in gingival tissues of human periodontitis in RT-qPCR (*n* = 30). **B** Correlation between *VNN1* and *IL1B* expression levels, and between *VNN2* and *IL1B* expression levels, in periodontitis in GSE10334 (*n* = 183). **C** Correlation between *VNN1* and *IL1B* expression levels, and between *VNN2* and *IL1B* expression levels, in periodontitis in GSE16134 (*n* = 241). Significance was defined in the figure as *** *p* < 0.001, and **** *p* < 0.0001

The relationship between *VNN1* and *VNN2*, and *IL1B*, an indicator of inflammation, was investigated based on datasets and RT-qPCR. As shown in Fig. 5, correlation analysis supported that *VNN1* expression was significantly positively correlated with *IL1B* in inflamed periodontal tissues by RT-qPCR (*n* = 30, R = 0.5911, *p* = 0.0006, moderate), and in the datasets GSE10334 (*n* = 183, R = 0.4208, *p* < 0.0001, moderate), and GSE16134 (*n* = 241, R = 0.3932, *p* < 0.0001, weak). For *VNN2*, positive correlations were also shown in the tissue samples (*n* = 30, R = 0.7651, *p* < 0.0001, strong) and datasets, GSE10334 (*n* = 183, R = 0.6477, *p* < 0.0001, moderate), and GSE16134 (*n* = 241, R = 0.6207, *p* < 0.0001, moderate). All these results indicated a potential association between Vanin family members and periodontitis.

### Potential contribution of VNN2 to periodontitis through neutrophils

**Fig. 6:**
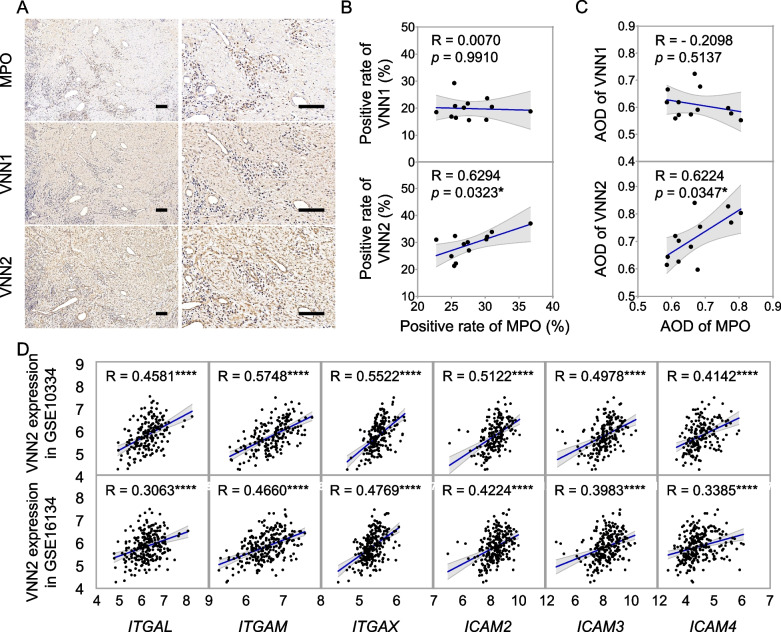
Upregulated VNN2 was correlated with neutrophil-related immune response in gingival tissues with periodontitis. **A** Immunohistochemical staining of MPO, VNN1 and VNN2 in gingival tissues with periodontitis. Positive expression areas of VNN1 and VNN2 were brownish yellow in the connective tissue. Positive expression of MPO was nucleus staining. Scale bar = 100 μm. **B** Scatter plot showed correlation between positive rate of MPO and that of VNN1 and VNN2 in diseased gingival tissues from periodontitis patients (*n* = 12). **C** The Scatter plot showed correlation between AOD of MPO and that of VNN1 and VNN2 in inflamed gingival tissues (*n* = 12). **D** Scatter plot showed correlation between *VNN2* gene expression and indicators of neutrophil adherence and migration in diseased gingival tissues of periodontitis in two datasets, GSE10334 and GSE16134. Significance was defined in the figure as * *p* < 0.05, and **** *p* < 0.0001. AOD, average optical density

Myeloperoxidase (MPO) expression was measured by IHC (Fig. 6A) in inflamed gingival tissues from periodontitis patients (*n* = 12). As shown in Fig. 6B, there was a significant and positive correlation between the positive rate of VNN2 and MPO (R = 0.6294, *p* = 0.0323, moderate). The AOD of VNN2 was also significantly positively correlated to MPO (R = 0.6224, *p* = 0.0347, moderate) in Fig. 6C. No significant correlation was observed between MPO and VNN1 expression (Fig. 6B, 6C).

To further explore the correlation between VNN2 and neutrophils, five genes of integrins, *ITGB2/CD18*, *ITGAL/CD11a*, *ITGAM/CD11b*, *ITGAX/CD11c*, and *ITGAD/CD11d,* and 14 genes of 12 ligands, JAM-1, JAM-3, ICAM‑1, ICAM‑2, ICAM‑3, ICAM‑4, ICAM-5, Laminin 8, uPAR, RAGE, Fibrinogen, and VCAM-1 were enrolled as indicators of β2 integrin-dependent neutrophil adherence and migration. As presented in Table 4 and Fig. 6D. The correlation analysis showed the positive association between *VNN2* expression level and indicators of neutrophil adherence and migration, including *ITGAL*, *ITGAM*, *ITGAX*, *ICAM2*, *ICAM3* and *ICAM4*, in periodontitis in both two datasets (R = 0.40–0.69, *p* < 0.0001, moderate). Taken together, VNN2 was suggested to potentially influence the inflammatory response via regulating β2 integrin-dependent neutrophil adherence and migration in periodontitis.

**Table 4:** Correlation between *VNN2* and indicators of β2 integrin-dependent neutrophil adherence and migration in periodontitis

Gene	Type		GSE10334 (*n* = 183)		GSE16134 (*n* = 241)	
			R	*p*	R	*p*
*ITGB2/CD18*	Integrin	β2	0.3993****	< 0.0001	0.2649****	< 0.0001
*ITGAL/CD11a*	Integrin	αL	0.4581****	< 0.0001	0.3063****	< 0.0001
*ITGAM/CD11b*	Integrin	αM	0.5748****	< 0.0001	0.4660****	< 0.0001
*ITGAX/CD11c*	Integrin	αX	0.5522****	< 0.0001	0.4769****	< 0.0001
*ITGAD/CD11d*	Integrin	αD	0.0643	0.3874	0.0358	0.5799
*ICAM1*	Ligand	ICAM-1	0.0845	0.2556	0.0371	0.5666
*ICAM2*	Ligand	ICAM-2	0.5122****	< 0.0001	0.4224****	< 0.0001
*ICAM3*	Ligand	ICAM-3	0.4978****	< 0.0001	0.3983****	< 0.0001
*ICAM4*	Ligand	ICAM-4	0.4142****	< 0.0001	0.3385****	< 0.0001
*ICAM5*	Ligand	ICAM-5	0.0623	0.4022	0.0266	0.6813
*F11R*	Ligand	JAM-1	-0.3305****	< 0.0001	-0.2903****	< 0.0001
*JAM3*	Ligand	JAM-3	0.0734	0.3233	0.0103	0.8733
*AGER*	Ligand	RAGE	-0.0695	0.3499	-0.1317*	0.0411
*FGA*	Ligand	Fibrinogen	0.0694	0.3507	0.1180	0.0674
*FGB*	Ligand	Fibrinogen	0.1198	0.1063	0.0721	0.2647
*FGG*	Ligand	Fibrinogen	-0.2677***	0.0002	-0.2179***	0.0007
*PLAUR*	Ligand	uPAR	0.2739***	0.0002	0.2040**	0.0015
*LAMC1*	Ligand	Laminin 8	0.3070****	< 0.0001	0.2647****	< 0.0001
*VCAM1*	Ligand	VCAM1	0.2536***	0.0005	0.2056**	0.0013

Statistical significance was indicated as * *p* < 0.05, ** *p* < 0.01, *** *p* < 0.001 and **** *p* < 0.0001

## Discussion

In the current study, we analyzed the expression profile of Vanin family members and their correlation with periodontitis on the aspect of clinical parameters, the severity of inflammation and neutrophil response. Results suggested that the Vanins were upregulated in the gingival tissues from patients with periodontitis when compared with periodontal healthy individuals, among which two members, VNN1 and VNN2 were elevated on both the genetic and protein levels. Correlation analyses further demonstrated that VNN1 and VNN2 were respectively associated with clinical parameters, PD and CAL, and indicator of inflammation, *IL-1B*. What’s more, the VNN2 might interact with neutrophil during periodontitis pathogenesis, with positive evidence between VNN2 and neutrophil-related biomarkers, MPO and indicators of β2 integrin-dependent neutrophil adherence and migration. This study characterized the expression profile of Vanins family members and their contribution to periodontitis.

For clinical relevance, Vanins have been closely related to clinical indicators in diseased animal models, and other human samples, alone with the conclusion that the abnormal accumulation of Vanins may lead to decreased biological function and prognosis [19–21, 30–33]. For instance, plasma VNN1 is increased in trauma patients and is independently associated with the risk of sepsis [20]. For patients with rheumatoid arthritis, VNN2 presents high concentrations in synovial fluids, indicating its role in inflammatory disease [21]. Vanins are also associated with decreased kidney function on the aspect of worse clinical parameters of renal function in rat model and human beings [31–33]. The clinical significance of Vanins provides clues for further studies about diagnosis and prognosis of diseases. In the current study, we performed comparison of Vanins expression between periodontitis and periodontal healthy gingival tissues, and concluded that higher concentrations of Vanins were accompanied with worse periodontal health conditions. With the primary aim to create accessibility for deep untreated pockets, periodontal surgery should be limited to periodontal pockets deeper than 5 mm to avoid mechanical damage to the periodontium[34]. In this study, gingival tissue samples, from patients with PD = (6.75 ± 1.41) mm and CAL = (6.06 ± 2.04) mm, were collected during periodontal flap surgery. The results may be more valid among the patients with severe periodontitis due to the sample collection methods. In the future, periodontitis of different severity, from mild to severe, could be recruited to better demonstrate the relationship between Vanins and this inflammatory disease, and to improve the generalizability of the findings.

In this study, correlation analysis was conducted between Vanins and *IL1B*, which is a highly inflammatory cytokine and promotes the recruitment of neutrophils at the inflamed sites through the overexpression of E-selectin and ICAM-1 [35–37]. NLRP3 inflammasome-dependent IL-1β production was reported to promote neutrophil recruitment, and neutrophils were in turn important sources of IL-1β in acute inflammatory disorders [38, 39]. Due to close relationships among IL-1β, neutrophils, and severity of inflammation, the positive correlation between Vanins and *IL1B* presented the potential functions of affecting the severity of the disease.

The mechanism behind Vanins in inflammation could be closely related to the regulation of neutrophil, including neutrophil activation, neutrophil degranulation, and leukocyte adhesion [40]. Previous studies have suggested that VNN2 regulates leukocyte adherence and migration through physically associating with Mac-1 (CD11b/CD18), an adhesive molecule essential to neutrophil function [41, 42]. In colorectal carcinogenesis, both Vanins and MPO interact with Mac-1 (CD11b/CD18), and regulate tissue destruction by activating inflammatory cells to the inflamed site [43]. In the current study, the positive correlation between VNN2 and MPO in IHC presented the potential regulation of VNN2 during this process. The correlation analysis between VNN2 and indicators of β2 integrin-dependent neutrophil adherence and migration further provided evidence to the contribution of VNN2 through regulating neutrophil migration by associating with Mac-1 (CD11b/CD18) on the human neutrophil surface in periodontitis.

On the other hand, VNN1 was not correlated to MPO in IHC, which led us to think further about the possible mechanism. It is well known that VNN1 could influence oxidative stress response and license the production of inflammatory mediators by antagonizing peroxisome proliferator-activated receptor-γ (PPAR-γ), which works as a negative regulatory factor of NF-κB with anti-inflammatory effects [44]. The absence of VNN1 could reduce inflammation and oxidative stress during infection or injury [45]. In acute pancreatitis, upregulation of S100A9 induces cell injury and inflammatory response through activating NLRP3 via targeting VNN1-mediated ROS release [15]. Considerable evidence has implicated the association of reactive oxygen response with the pathogenesis of periodontitis [10, 46]. The inflammatory response in periodontitis was suggested to be associated with increased local and systemic oxidative stress and impaired antioxidant capacity [4, 10, 47]. The link between periodontitis, oxygen stress and VNN1 may provide clues for further exploration of the pathological process of periodontitis [10]. Therefore, further studies need to be carried out to investigate the deeper mechanism behind Vanins’ regulation in periodontitis.

## Conclusion

In summary, this study revealed the upregulation of Vanin family members in periodontitis at both genetic and protein levels. The clinical significance of Vanins and their positive association with inflammation indicated their potential involvement in periodontitis. Furthermore, VNN2 was positively correlated with indicators of neutrophils, indicating the potential contribution of VNN2 to periodontitis via affecting neutrophil adherence and migration during immune response. All our results indicated possible significance of Vanin family members in human periodontitis and might act as potential therapeutic targets to treat periodontitis.

## Supplementary Information

**Additional file 1**: **Supplementary File 1**. STROBE checklist. The study has followed the STROBE guidelines and added the STROBE checklist.

**Additional file 2**: **Supplementary Figure 1**. Flow diagram for the study groups. The enrollment of participants for the periodontitis group (A) and the periodontal healthy group (B) were presented respectively. **Supplementary Figure 2**. Uncropped images of western blot in Figure 3, including bolts of VNN1 (A), VNN2 (B), and GAPDH (C). P, periodontitis group; H, periodontal healthy group.

## Data Availability

Data will be available from corresponding authors on the reasonable request.

## Abbreviations

AOD: Average optical density
BOP: Bleeding on probing
CAL: Clinical attachment loss
GEO: Gene Expression Omnibus
HE: Hematoxylin–eosin
IHC: Immunohistochemistry
MPO: Myeloperoxidase
NCBI: National Center for Biotechnology Information
PPAR-γ: Peroxisome proliferator-activated receptor-γ
PBS: Phosphate buffered saline
PD: Probing depth
RT-qPCR: Real-time quantitative polymerase chain reaction
SD: Standard deviation
VNN: Vanin
