# Supplementary figures and images for: Upregulated Vanins and their potential contribution to periodontitis
Source: BMC Oral Health. 2022 Dec 17;22:614. doi: 10.1186/s12903-022-02583-7 (PMC9758802; doi:10.1186/s12903-022-02583-7)

# Supplementary Figure 1

A

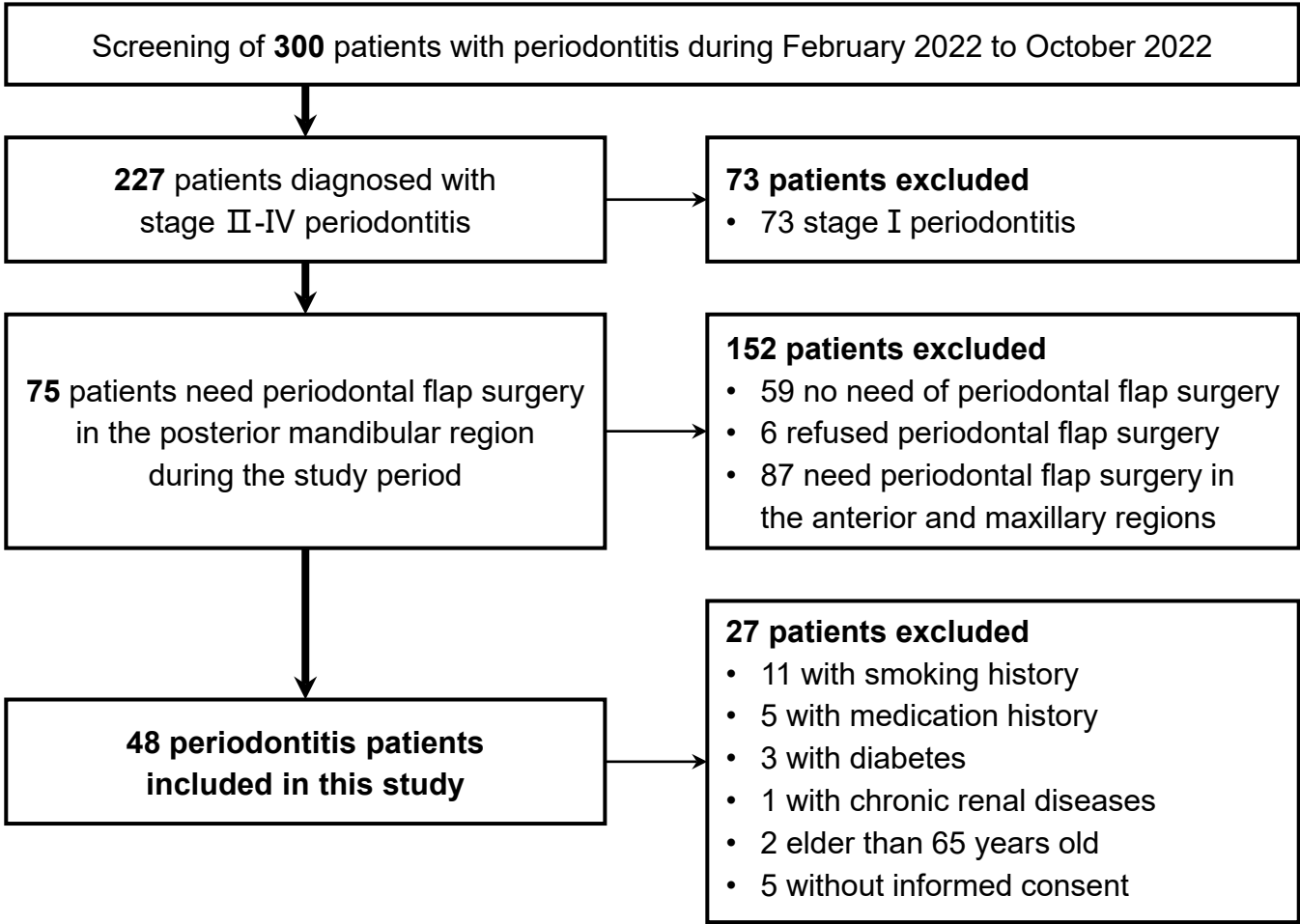

B

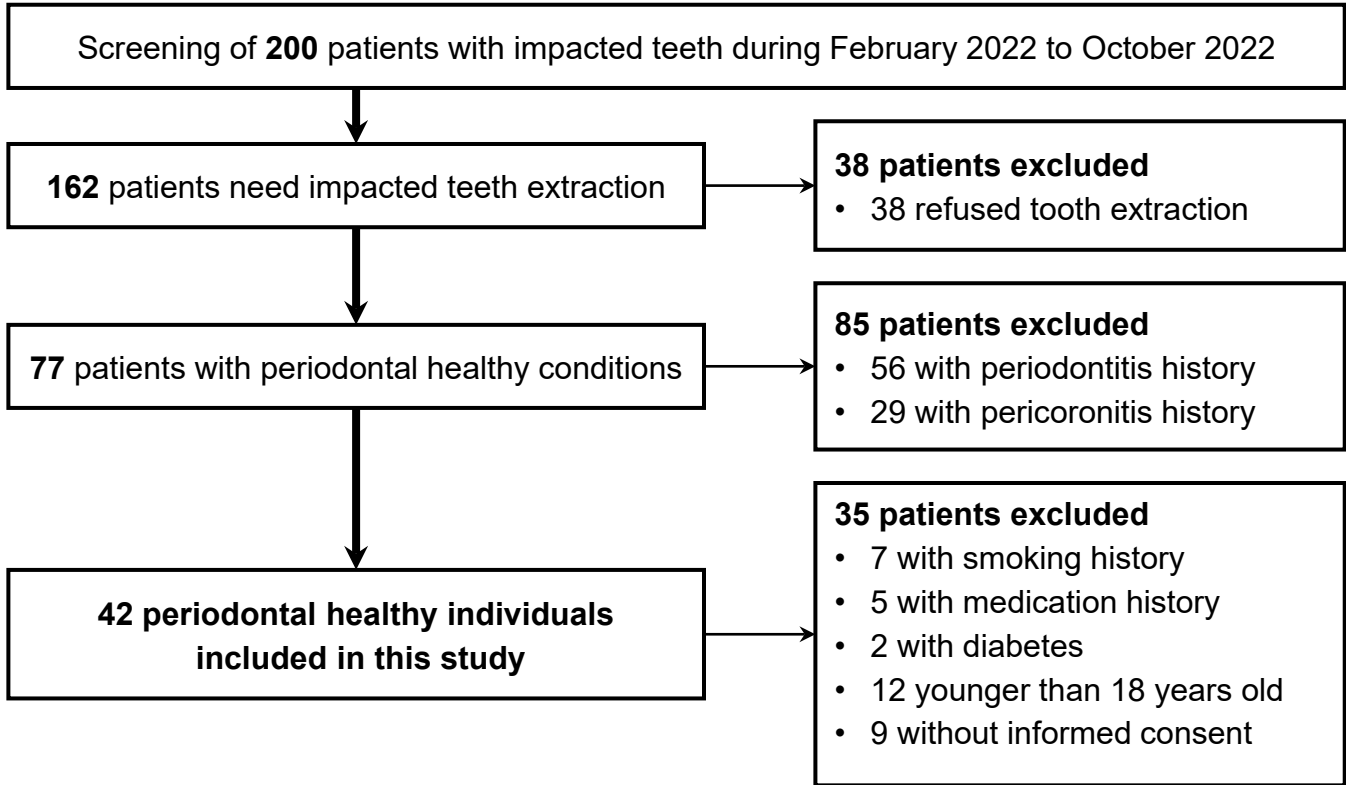

Supplementary Figure 2

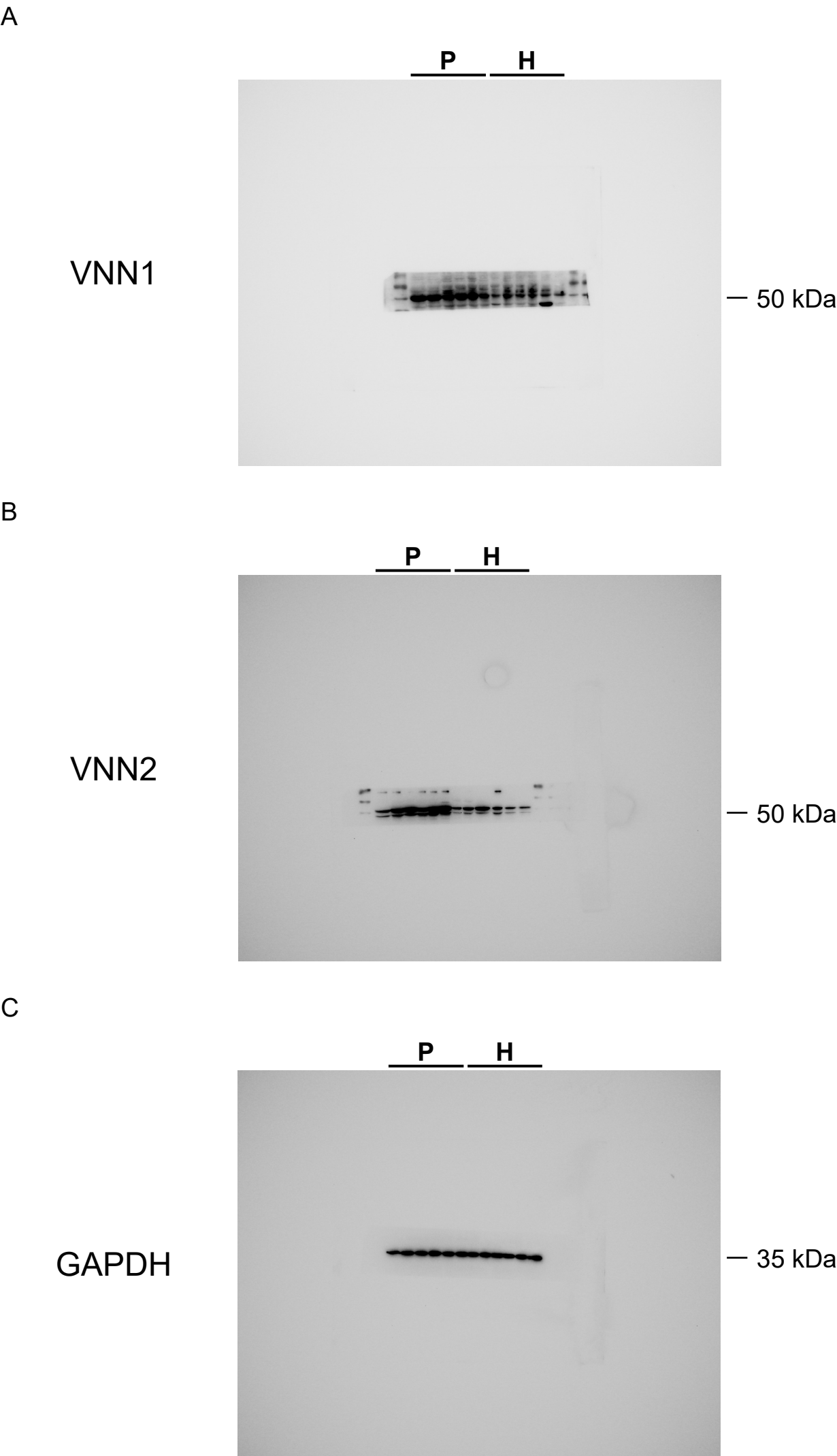

Supplement: Supplementary file 2 — Additional file 2: Supplementary Figure 1. Flow diagram for the study groups. The enrollment of participants for the periodontitis group (A) and the periodontal healthy group (B) were presented respectively. Supplementary Figure 2. Uncropped images of western blot in Figure 3, including bolts of VNN1 (A), VNN2 (B), and GAPDH (C). P, periodontitis group; H, periodontal healthy group. [file 12903_2022_2583_MOESM2_ESM.pdf]
